# Transcriptional and Post-Transcriptional Modulation of SPI1 and SPI2 Expression by ppGpp, RpoS and DksA in *Salmonella enterica* sv Typhimurium

**DOI:** 10.1371/journal.pone.0127523

**Published:** 2015-06-03

**Authors:** Christopher J. Rice, Vinoy K. Ramachandran, Neil Shearer, Arthur Thompson

**Affiliations:** 1 Institute of Food Research, Norwich, NR4 7UA, United Kingdom; 2 Department of Plant Sciences, University of Oxford, Oxford, OX1 3RB, United Kingdom; Indian Institute of Science, INDIA

## Abstract

The expression of genes within *Salmonella* Pathogenicity Islands 1 and 2 (SPI1, SPI2) is required to facilitate invasion and intracellular replication respectively of *S*. Typhimurium in host cell lines. Control of their expression is complex and occurs via a variety of factors operating at transcriptional and post-transcriptional levels in response to the environmental stimuli found within the host. Several of the factors that modulate SPI1 and SPI2 expression are involved in the redistribution or modification of RNA polymerase (RNAP) specificity. These factors include the bacterial alarmone, ppGpp, the alternative sigma factor, RpoS, and the RNAP accessory protein, DksA. In this report we show not only how these three factors modulate SPI1 and SPI2 expression but also how they contribute to the ‘phased’ expression of SPI1 and SPI2 during progress through late-log and stationary phase in aerobic rich broth culture conditions. In addition, we demonstrate that the expression of at least one SPI1-encoded protein, SipC is subject to DksA-dependent post-transcriptional control.

## Introduction

Pathogenic serovars of *Salmonella* infect both humans and animals, causing in humans either a self-limited gastroenteritis (e.g. *S*. Typhimurium, *S*. Enteriditis), or potentially fatal systemic infections (e.g. *S*. Typhi, *S*. Paratyphi). *Salmonella* is the second most reported zoonotic infection in humans and the most frequent cause of food borne outbreaks in the EU [1]. During infection, *Salmonella* invades epithelial cells lining the small intestine, mediated by *Salmonella* Pathogenicity Island 1 (SPI1), encoding a type 3 secretion system (T3SS). SPI1 triggers the injection of effector proteins into the host cell to facilitate uptake of bacteria during the process of invasion. Intracellular *Salmonella* employ a second T3SS encoded within SPI2, which modifies the initial membrane-bound compartment or phagosome to form the ‘*Salmonella* containing vacuole’ (SCV) [2]. The SCV avoids fusion with lysosomes, enabling *Salmonella* to evade the antimicrobial compounds that form part of the host immune response. In systemic infections, *Salmonella* passes through the gut wall and is phagocytosed by macrophages which can transport and disseminate the pathogen throughout the host [3,4].

One of the major regulators of virulence gene expression in *Salmonella* is the bacterial alarmone guanosine tetraphosphate (ppGpp) [5]. Using both microarray-based and differential RNA sequencing (dRNA-seq) approaches, it has been shown that ppGpp is required for the expression of nearly all of the genes within SPI1 and SPI2 as well as many other *Salmonella*-virulence related genes [5–8]. In addition, next-generation transcriptomics has revealed that ppGpp is involved in regulating several of the virulence-related, non-coding RNAs discovered thus far in *S*. Typhimurium [7]. Guanosine tetraphosphate is synthesised by the RelA and SpoT enzymes in all beta- and gammaproteobacteria and, whereas RelA only has ppGpp synthetic function, SpoT is able to both synthesise and hydrolyse ppGpp (for reviews see [9–11]). In addition to *Salmonella*, it has also been shown that ppGpp plays a key role in coupling virulence to metabolic status in several pathogenic bacteria including *Mycobacterium tuberculosis* [12,13], *Listeria monocytogenes* [14], *Legionella pneumophilia* [15,16], *Vibrio cholera* [17] and *Pseudomonas aeruginosa* [18].

DksA is a small 151 amino acid protein found in most bacterial species, including *S*. Typhimurium and *Escherichia coli*. DksA was originally discovered as a dose dependent suppressor in a *dnaK* deletion mutant. Subsequently, DksA was found to play a physiologically pleiotropic role including mediating chaperonin function, cell division, amino acid biosynthesis, phage sensitivity, quorum sensing, responses to envelope stress and virulence [19,20]. DksA is thought to mediate these effects via directly binding to RNA polymerase (RNAP). As a consequence of this mechanism of DksA binding, RNAP is sensitive to changes in ppGpp concentration (and the initial NTP of the transcript), resulting in the reduction or inhibition of rRNA transcription at low steady state growth rates and during entry into stationary phase [20]. In addition to inhibiting some promoters, ppGpp and DksA can also activate promoters through a direct and/or indirect mechanism [21–25]. Indirect activation may occur via liberation of RNAP from rRNA operons, thereby increasing its availability to lower affinity promoters or promoters that are able to make higher-stability complexes with RNAP. DksA and ppGpp also indirectly regulate several promoters that are transcribed by alternative sigma factors (e.g. σ^54^ and σ^S^). This regulation has been suggested to occur either as a result of competition for RNAP, by alternative sigma factors, or through some other mechanism [26,27]. As well as the above, it has been shown that the zinc finger motif of DksA can serve as a thiol switch to sense oxidative and nitrosative stress, which may suggest one reason why *S*. Typhimurium *dksA* mutants are attenuated in mouse infection models [28,29]. Finally, in addition to *Salmonella*, virulence regulation has been attributed to DksA in *P*. *aeruginosa*, *S*. *flexneri*, and *E*. *coli*, and to a DksA-like protein in *C*. *jejuni* [22,25,30–33].

The alternative sigma factor, RpoS (σ^S^_,_ σ^38^) is involved in the general stress response, and is induced during entry into stationary phase (for review, see [34]). Production of RpoS occurs very rapidly upon entry into stationary phase but protein concentrations are maintained at very low levels in exponentially growing cells. Regulation of RpoS occurs at multiple levels—transcription, translation, degradation and activity; the large number of stresses that are transduced via RpoS occur at one or more of these regulatory levels. RpoS is involved in the virulence mechanisms of many bacterial pathogens; however its effect on virulence appears to be variable. RpoS has been found to be necessary for virulence in certain pathogens including *Salmonella enterica*, *Vibrio cholerae*, *Burkholderia plantarii*, and *Serratia entomophila* but less important in other pathogens [35–40], reviewed in [41]. In this study, we determine the roles of three RNAP modulatory elements, ppGpp, RpoS and DksA, in the control of SPI1 and SPI2 expression during stationary phase in aerated rich broth culture. Whereas ppGpp activates SPI1 and SPI2 expression at different points during stationary phase, RpoS reduces their expression, and DksA can act as both a repressor and an activator of SPI1 and SPI2 encoded genes respectively. The disparate effects of ppGpp, RpoS and DksA on SPI1 and SPI2 expression suggests they may play a role in controlling the often mutually exclusive expression of these pathogenicity islands during invasion or intracellular growth [42].

## Results

### ppGpp and RpoS contribute to the modulation of SPI1 and SPI2 transcript levels during stationary phase

**Fig 1:**
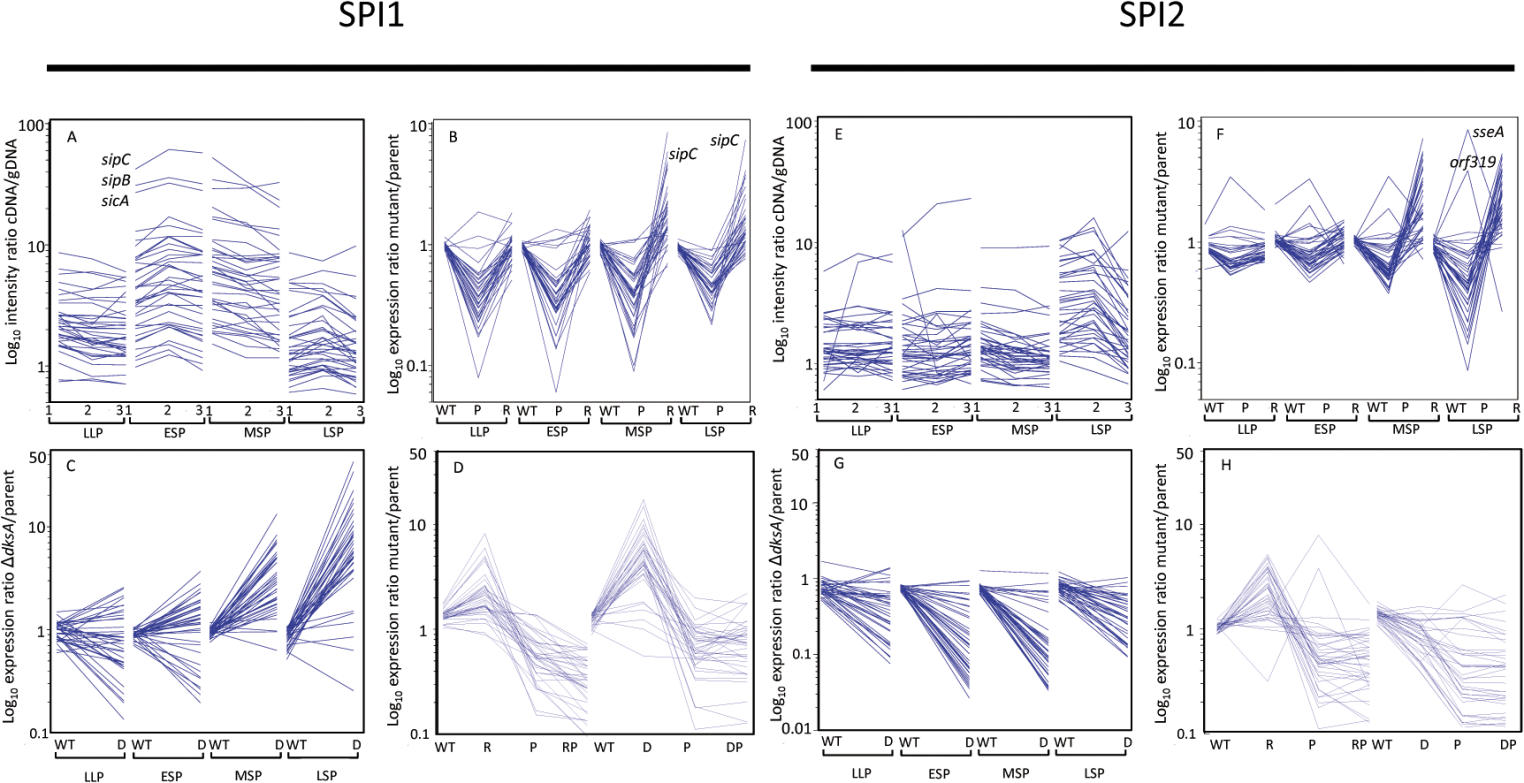
Effect of loss of ppGpp, Rpos and/or DksA on SPI1 and SPI2 expression at late-log phase (LLP), early stationary phase (ESP), mid-stationary phase (MSP) and late stationary phase (LSP). (A, E) SPI1 and SPI2 transcript levels in parent strain (SL1344); 1, 2, and 3 indicate biological replicate cultures. (B, F) SPI1 and SPI2 transcript levels in Δ*relA*Δ*spoT* (P) and Δ*rpoS* (R) strains; transcript levels are normalised to parental (WT) SPI1 transcript levels. (C, G) SPI1 and SPI2 transcript levels in a Δ*dksA* (D) strain normalised to parent (WT) strain. (D, H) Late stationary phase SPI1 and SPI2 transcript levels in Δ*relA*Δ*spoT* (P), Δ*rpoS* (R), and Δ*relA*Δ*spoT*Δ*rpoS* (RP) and Δ*relA*Δ*spoT*Δ*dksA* (DP) strains normalised to transcript levels in the SL1344 parent strain. Data from which the figure was compiled and statistical analysis is shown in S2 Fig and also deposited at Gene Expression Omnibus (GEO), superseries accession number GSE63715.

**Fig 2:**
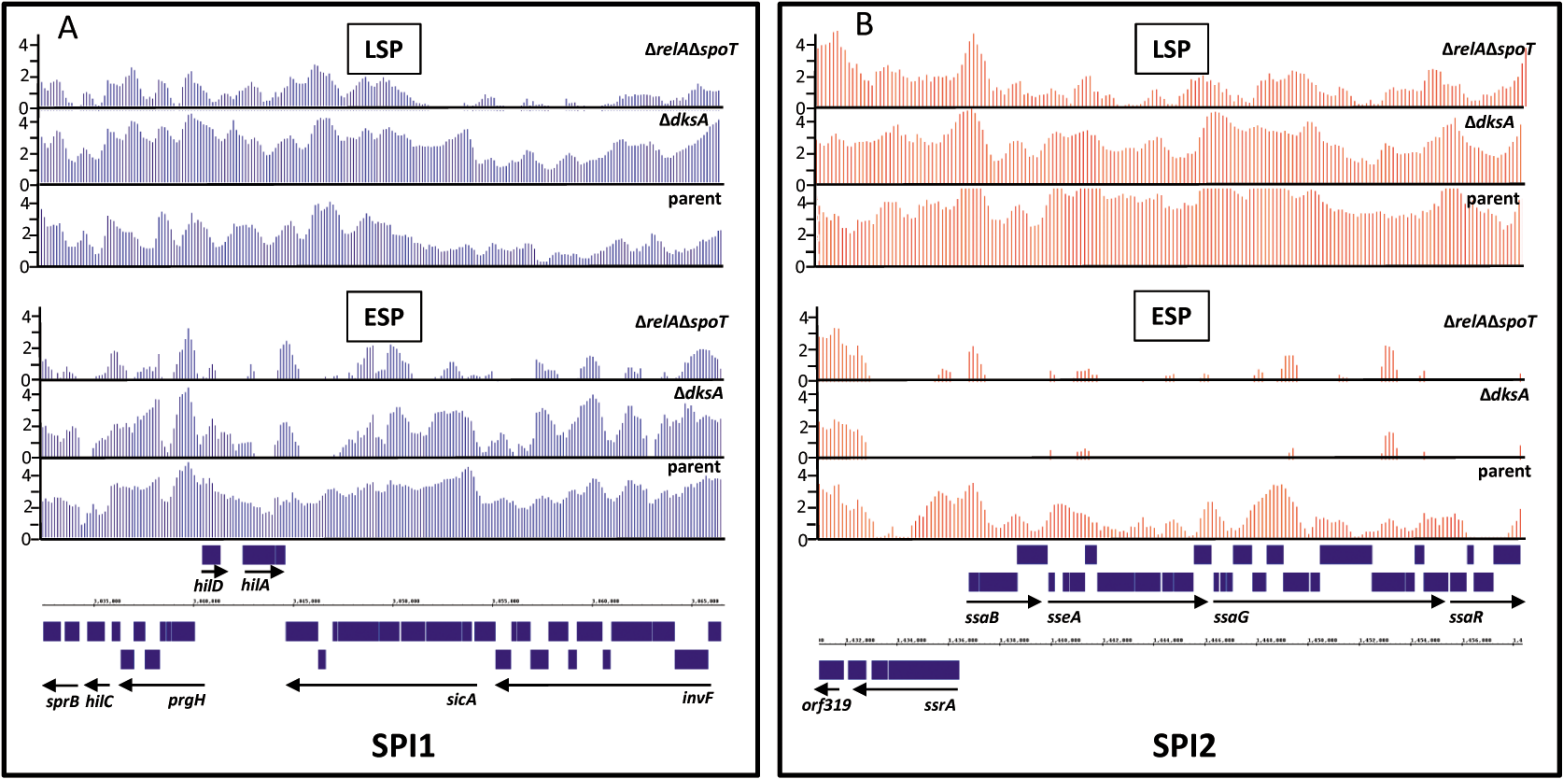
ChIP-chip profiles of RNAP distribution at the (A) SPI1 and (B) SPI2 loci in parental and Δ*dksA* and Δ*relA*Δ*spoT* strains at ESP and LSP viewed on the integrated genome browser (IGB) [65]. Resolution of each peak is 100 nt.

During progress through late-log and stationary phase, batch cultures of *S*. Typhimurium growing aerobically in Luria-Bertani (LB) medium express SPI1 and SPI2 encoded genes (Fig 1A and 1E) [5, 6, 43]. *S*. Typhimurium cultures at the late-log stage of growth are frequently used to promote invasion of epithelial cell lines to determine intracellular replication rates in tissue-culture based gentamicin protection assays. According to microarray-based transcriptomic analyses, SPI1 gene expression increases and is maximal at an OD_600_ of 2.3 to 3.0, thereafter expression declines, a finding in accordance with previous work [43]. In the present study, during early stationary phase (ESP; OD_600_ = 2.3), the most highly expressed SPI1 genes were found to be *sipC*, *sipB* and *sicA* (Fig 1A). During later stationary phase (OD_600_ = 4.2), microarray (Fig 1E, [6]), ChIP-chip data (Fig 2B) and dRNA-seq data [43] show that SPI2 expression increases. SPI1 and SPI2 expression is complex and controlled by a number of different factors that operate at the transcriptional and post-transcriptional levels, and that respond to environmental cues. However, one of the major signals for the induction of SPI1 and SPI2 expression in response to environmental conditions, both *in vitro* and *in vivo* is the bacterial alarmone, ppGpp [5–8,44]. Both microarray and dRNA-seq data showed that in the absence of ppGpp (Δ*relA*Δ*spoT*), SPI1 and SPI2 transcript levels are extremely low compared to the parent strain (Fig 1B and 1F, S2 Table, [7,43]). Since ppGpp acts primarily to redistribute RNA polymerase, the very low levels of SPI1 and SPI2 transcription in the absence of ppGpp strongly suggested that there was a lack of RNAP recruitment at SPI1 and SPI2 sites in the Δ*relA*Δ*spoT* strain. A ChIP-chip analysis using an antibody to the beta subunit of *E*. *coli* RNAP verified that this was indeed the case (Fig 2A and 2B). Interestingly, the expression of a few SPI2 genes increased in the Δ*relA*Δ*spoT* compared to the parent strain, suggesting they are ppGpp-repressed; these were *orf319* (4.4-fold) and *sseA* (10.3-fold), (S2 Table, Fig 1F).

In addition to SPI1 and SPI2, the alternative sigma factor, RpoS is known to be highly expressed during stationary phase in *S*. Typhimurium and has been shown to be required for the successful infection of mice. We therefore decided to investigate the effect of RpoS on SPI1 and SPI2 expression. Using a strain of *S*. Typhimurium in which the *rpoS* gene had been deleted, we found that the transcript levels of SPI1 encoded genes at late log phase (LLP) and ESP were very similar (less than 2–fold) compared to the parent strain (S2 Table). However, expression of the *sicA* operon (consisting of the virulence factors *sicA*, *sipB*, *sipC*, *sipD*, and *sipA*) was significantly elevated by up to 8-fold at mid- and late-stationary phases; MSP and LSP (Fig 1B, S2 Table); in addition *sicP*, *STM2880* and *hilA* were significantly elevated > 2-fold at LSP (S2 Table). We also observed a similar elevation of SPI2 transcript levels in the Δ*rpoS* compared to the parent strain at MSP and LSP, but not at LLP and ESP (Fig 1F). The SPI2 encoded genes significantly elevated by > 2-fold at both MSP and LSP were *ssaCGHIJKLNO*, *sscAB*, *sseCDEG* and the SPI2 regulators, *ssrAB*; additionally, *ssaBJMR* and *ssaT* were also significantly elevated between 2 and 4.2-fold at LSP (S2 Table).

**Fig 3:**
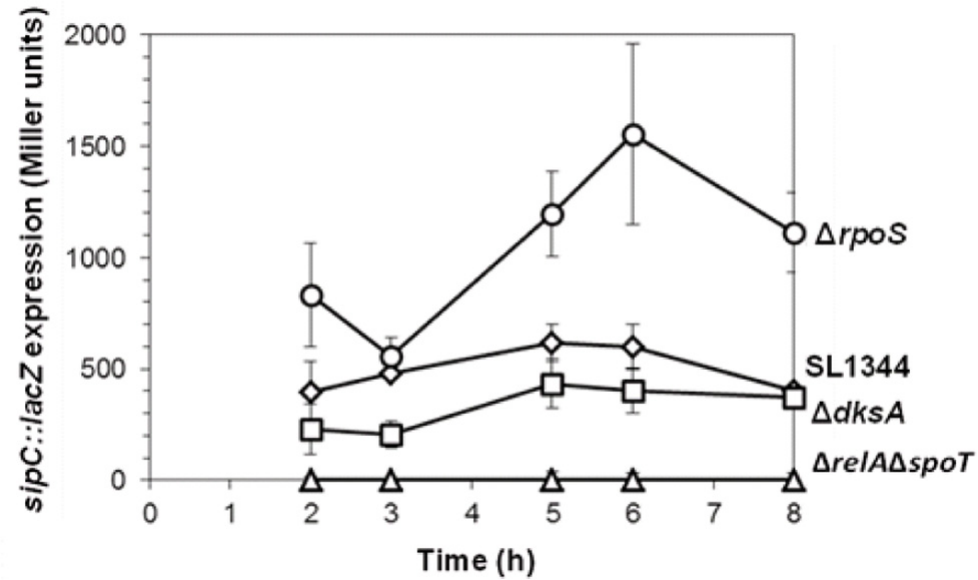
Expression of an *S*. Typhimurium *sipC*::*lacZ* fusion in parental, Δ*relA*Δ*spoT* (ppGpp^0^), Δ*dksA* and Δ*rpoS* backgrounds during growth in aerobic LB batch cultures. Data is from 3 biological replicate experiments.

**Fig 4:**
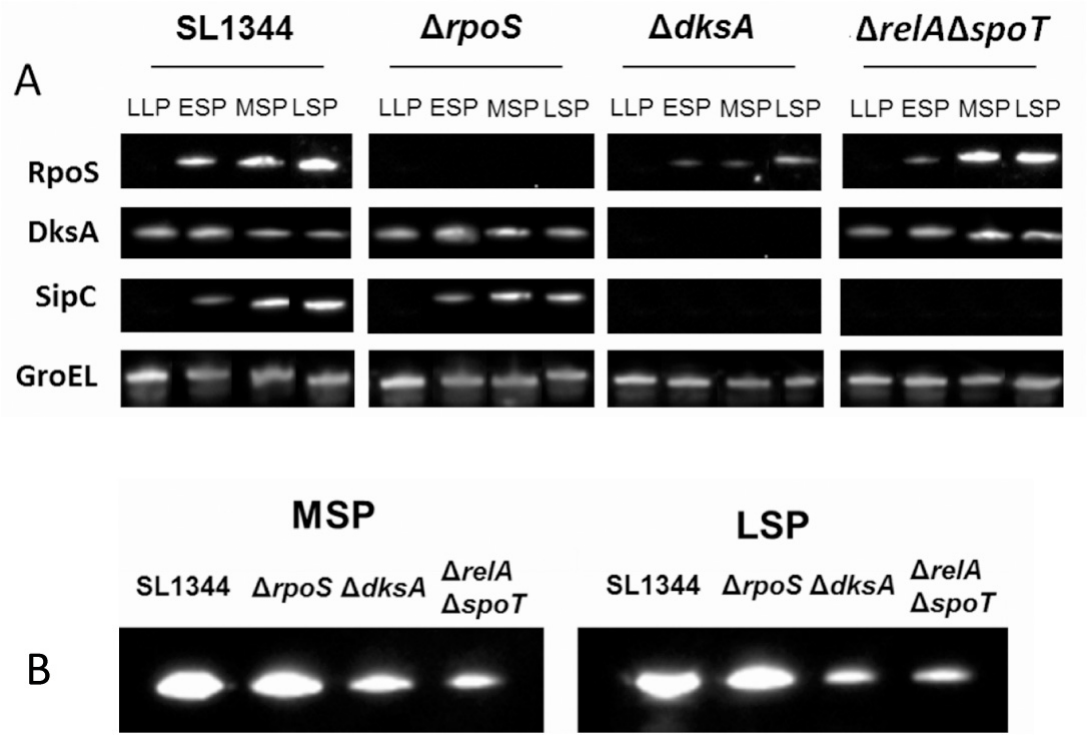
(A) western blot showing intracellular levels of RpoS, DksA, SipC and GroEL (loading control) at LLP, ESP, MSP and LSP in parental (SL1344), Δ*rpoS*, Δ*dksA* and ppGpp^0^ (Δ*relA*Δ*spoT*) strains. (B) western blot showing SipC levels in culture supernatants from parental, Δ*rpoS*, Δ*dksA* and Δ*relA*Δ*spoT* strains during mid stationary phase (MSP) and late stationary phase (LSP).

**Fig 5:**
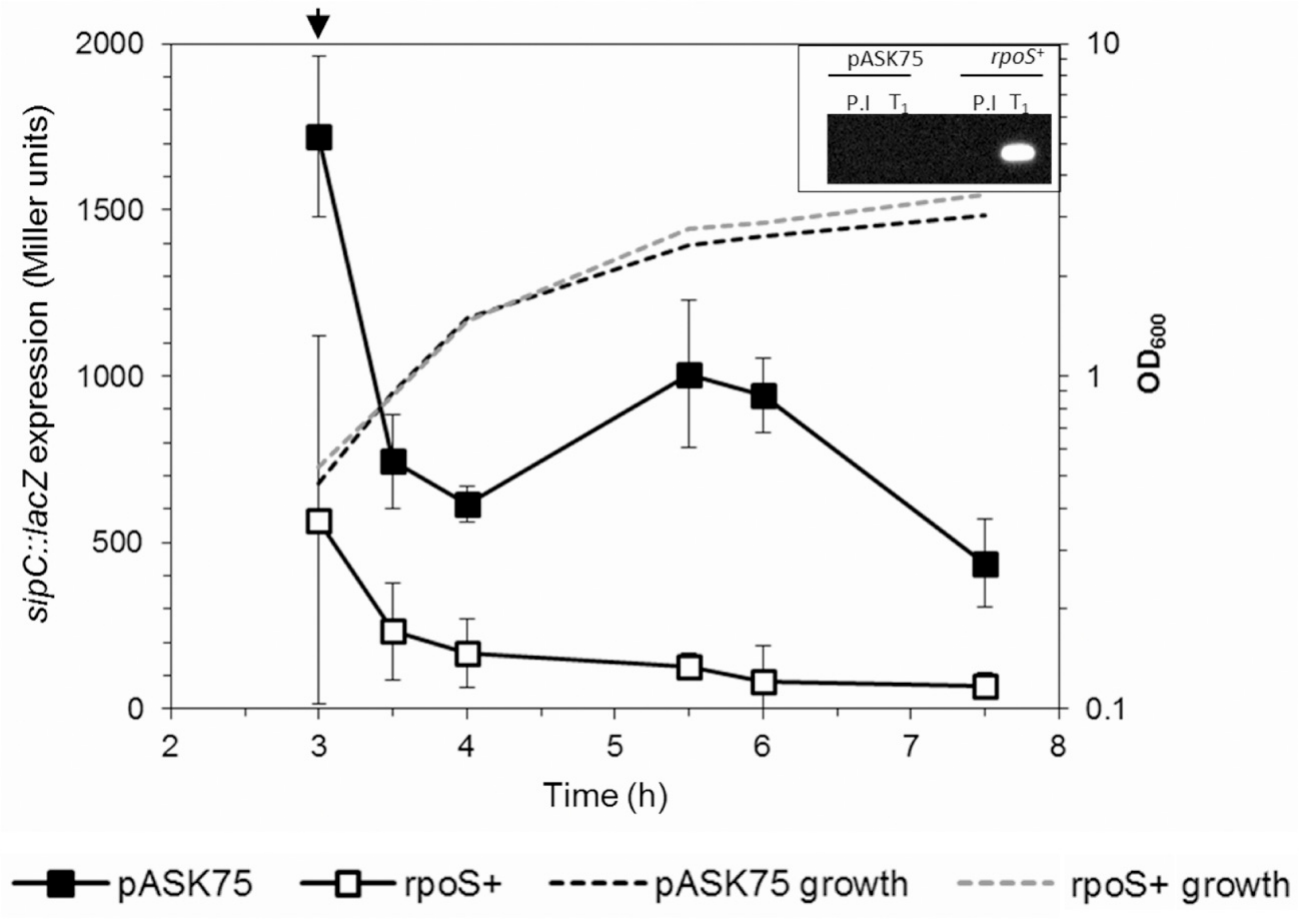
Effect of RpoS over-expression on *sipC*::*lacZ* activity during growth in LB batch culture. Inset: western blot showing absence of RpoS immediately prior to induction (P.I), and following 1h induction with 0.2 μg.ml^**-1**^ anhydrotetracycline. The OD_600_’s were 0.626 and 0.664 for pre-induction cultures containing the pASK75 empty vector and the *rpoS* ^**+**^ vector respectively. Following induction for 1h with ATC, the OD_600_’s were 1.54 and 1.73 respectively for cultures containing the pASK75 empty vector and the *rpoS* ^**+**^ vector.

Using *sipC* as an example of a SPI1 encoded gene exhibiting high transcript levels at MSP and LSP in the Δ*rpoS* compared to the parent strain (Fig 1A, S2 Table), we performed β-galactosidase assays on a *sipC*::*lacZ* construct in the Δ*rpoS* and parent strains to verify the elevated *sipC* transcript levels (Fig 3). This result showed that expression of a *sipC*::*lacZ* fusion increased during stationary phase, peaking at 3-fold higher activity levels in the Δ*rpoS* strain compared to the parent strain after 6h growth, corresponding to the mid to late stationary phase of growth. Interestingly, despite the observations of elevated *sipC* transcript levels in the Δ*rpoS* relative to the parent strain (5.7 and 7.0-fold respectively at MSP and LSP), and elevated *sipC*::*lacZ* expression in the Δ*rpoS* strain, western blots revealed little difference in intracellular or culture supernatant SipC protein levels in the Δ*rpoS* strain relative to the parent strain during stationary phase (Fig 4A and 4B). In addition, the relatively small changes in expression levels of the *sipC*::*lacZ* fusion in the parent strain during stationary phase (Fig 3), when compared to the significantly elevated *sipC* transcript levels observed in the parent strain at ESP and MSP (7.4 and 2.2-fold respectively, Fig 1A, S2 Table) is suggestive of post-transcriptional control of SipC stability. Despite our observation that *sipC*::*lacZ* activity increased 3-fold in the Δ*rpoS* strain through early and late stationary phase (Fig 3), controlled over-expression of *rpoS* from an inducible promoter resulted in a considerable decrease of *sipC*::*lacZ* activity compared to the control during mid- and late stationary phase (Fig 5). One explanation for this observation is that RpoS is able to compete for RNAP availability to reduce the ppGpp-dependent recruitment of RNAP and thus reduce *sipC* transcription; this would be consistent with the sigma factor competition model of RNAP distribution [44].

One prediction of the above model would be that the presence or absence of RpoS in a ppGpp^0^ (Δ*relA*Δ*spoT*) background would make little difference to SPI1 or SPI2 transcript levels, since RNAP could not be efficiently recruited to SPI promoters in the absence of ppGpp. In accordance with this, we found that the significantly elevated expression (> 2-fold) of SPI1 and SPI2 genes observed in the Δ*rpoS* strain relative to the parent did not occur in a comparison with the Δ*relA*Δ*spoT*Δ*rpoS* strain, and in fact the expression of the majority of SPI1 and SPI2 genes in the latter strain decreased to the similar levels observed in the Δ*relA*Δ*spoT* strain (Fig 1D and 1H, S2 Table). A few exceptions where transcript levels were higher in the Δ*relA*Δ*spoT* strain compared to the Δ*relA*Δ*spoT*Δ*rpoS* strain included *invF* (4.7-fold), *prgI* (4.0-fold), *invC* (2.1-fold) and *invJ* (2.0-fold), (S2 Table, Fig 1D). In a similar manner to SPI1, we found that within SPI2, the expression of only 4 genes showed significantly higher expression in the Δ*relA*Δ*spoT* strain compared to the Δ*relA*Δ*spoT*Δ*rpoS* strain; these were *orf319* (22.2-fold), *sseA* (6.1-fold), *ssaI* (2.1-fold) and *ssaG* (2.1-fold), (S2 Table, Fig 1H). Expression of the latter genes may therefore be RpoS as well as ppGpp-dependent.

Finally, in *E*. *coli* it has been shown that RpoS levels are positively controlled by ppGpp [45], however, in *S*. Typhimurium, RpoS levels appear to be unaffected by the absence of ppGpp [46]. Our data verified this observation in *S*. Typhimurium (Fig 4A). Whether these differences in control of RpoS by ppGpp in *S*. Typhimurium compared to *E*. coli represents an adaptation to optimising virulence gene expression relative to other RpoS-dependent regulatory requirements in *S*. Typhimurium remains to be seen.

### Regulation of SPI1 and SPI2 transcription by DksA during stationary phase

DksA is an RNAP accessory protein and has been shown to potentiate the effects of ppGpp [20,23,47]. Previous work has also demonstrated that DksA is required for virulence of *S*. Typhimurium in a murine infection model [32]. In order to investigate the role of DksA in SPI1 and SPI2 expression, we constructed an *S*. Typhimurium Δ*dksA* strain and showed that an early stationary phase culture of the mutant strain was attenuated by 3.6-fold for invasion in a HeLa cell infection model when compared to the parent strain (S4 Fig). We also performed a microarray-based transcriptomic analysis of the Δ*dksA* strain and showed that the transcript levels of 20 and 25 SPI1 encoded genes were significantly increased between 2 and 29-fold at MSP and LSP respectively compared to the parent strain (Fig 1C, S2 Table). The transcripts elevated in the Δ*dksA* strain at both MSP and LSP were *prgHIJK*, *invBCEFGHIJ*, *spaPR*, *iagB*, *STM2870*, *STM2891*, *orgA* and the SPI1 regulator, *hilD* (S2 Table). Consistent with the elevated expression of many SPI1 genes, a ChIP-chip analysis revealed an enhanced recruitment of RNAP to SPI1 promoter sites in the Δ*dksA* mutant compared to the parent strain at LSP (Fig 2A). In contrast to the elevated transcript levels of a majority of the SPI1 genes at MSP and LSP, the transcript levels of a subset of SPI1 genes were reduced at LLP and ESP by 2- to 5-fold (Fig 1C); these genes included all of those within the *sicA* operon (*sicA*, *sipBCDA*). The increased SPI1 transcript levels observed in the Δ*dksA* strain at LSP were found to be ppGpp-dependent since a Δ*relA*Δ*spoT*Δ*dksA* showed no significant increase of SPI1 transcript levels compared to the Δ*dksA* strain apart from *orgA* which was significantly reduced by 5.9-fold in the Δ*relA*Δ*spoT*Δ*dksA* compared to the Δ*relA*Δ*spoT* strain (S2 Table, Fig 1D). The latter data is consistent with a scenario where, in the absence of DksA (or a DksA-dependent transcription factor), recruitment of RNAP to SPI1 sites is ppGpp-dependently increased at LSP relative to the parent strain; this is corroborated by the ChIP-chip data which showed increased RNAP recruitment at SPI1 sites at LSP for the Δ*dksA* compared to the parent strain (Fig 2A). In the absence of ppGpp, recruitment of RNAP to SPI1 cannot efficiently occur (as the ChIP-chip data shows, Fig 2A), therefore the proposed effect of DksA (or a DksA-dependent transcription factor) in reducing recruitment or activity of RNAP is lost and SPI1 transcript levels in the Δ*relA*Δ*spoT*Δ*dksA* mutant become comparable to those in the Δ*relA*Δ*spoT* strain (Fig 1D). The observation that there was no discernible difference in DksA protein levels in the presence or absence of ppGpp is consistent with the above scenario (Fig 4A).

Although we observed elevated SPI1 transcript levels in the Δ*dksA* strain at MSP and LSP, we found that SPI2 transcript levels were reduced at all points sampled during late-log and stationary phase by up to 40-fold (Fig 1G, S2 Table). The observed reduction in SPI2 transcript levels in the Δ*dksA* strain during stationary phase correlated with a decreased recruitment of RNAP to SPI2 sites in the Δ*dksA* strain compared to the parent at ESP and LSP (Fig 2B). As expected, the distribution of RNAP to SPI2 genomic sites in the Δ*relA*Δ*spoT* strain was also reduced compared to the parent strain at both ESP and LSP, in accordance with the reduced SPI2 transcript levels observed in the former compared to the latter strain (Figs 1F and 2B). SPI2 transcript levels were also found to be further reduced in the Δ*relA*Δ*spoT*Δ*dksA* strain compared to the Δ*dksA* strain suggesting that DksA-dependent activation of their transcription requires ppGpp (Fig 1H, S2 Table). Interestingly, DksA therefore seems to have opposite effects on SPI1 and SPI2 transcription—on the one hand ppGpp-dependently repressing SPI1 transcription at MSP and LSP, (Fig 1C), whilst at the same time activating SPI2 expression (Fig 1G). The ChIP-chip data indicates these effects are likely to occur by modulating RNAP distribution (Fig 2).

### SipC levels are post-transcriptionally regulated by DksA

The transcript level of the SPI1 effector gene, *sipC* was found to be very high at ESP in the parent strain (7.4-fold compared to LLP, Fig 1A, S2 Table), and was also considerably elevated in the Δ*dksA* strain at LSP (4.9-fold compared to the parent strain, Fig 1C, S2 Table). We therefore decided to use western blotting to determine whether the level of SipC also changed in accordance with its transcript level in the Δ*dksA* mutant. When we tested the effect of loss of DksA on the intracellular and secreted levels of SipC, we were surprised to discover that intracellular SipC was undetectable at all of the time points sampled during late-log and stationary phase, despite the elevated *sipC* transcript levels observed at MSP and LSP (Fig 4A, S2 Fig). We were also unable to detect SipC in the Δ*relA*Δ*spoT* strain, however, this appears to be unrelated to the absence of SipC in the Δ*dksA* strain since DksA levels were unaffected in the Δ*relA*Δ*spoT* strain compared to the parent strain (Fig 4A). Despite the complete absence of intracellular SipC in the Δ*dksA* and Δ*relA*Δ*spoT* strains, SipC was detectable in culture medium, although at much lower amounts compared to the parent strain (Fig 4B); this may represent SipC accumulated from earlier growth phases. The observations that intracellular SipC was undetectable by western blotting, yet *sipC* transcripts were elevated at MSP and LSP according to the microarray and ChIP-chip data, and measurement of *sipC*::*lacZ* activity revealed only a slight decrease in the Δ*dksA* strain compared to the parent strain (Fig 3), suggests that the stability of SipC is regulated by a DksA-dependent post-transciptional mechanism during late-log/stationary phase.

### RpoS and DksA dependent expression levels of known SPI1 and SPI2 regulators at ESP and LSP

**Fig 6:**
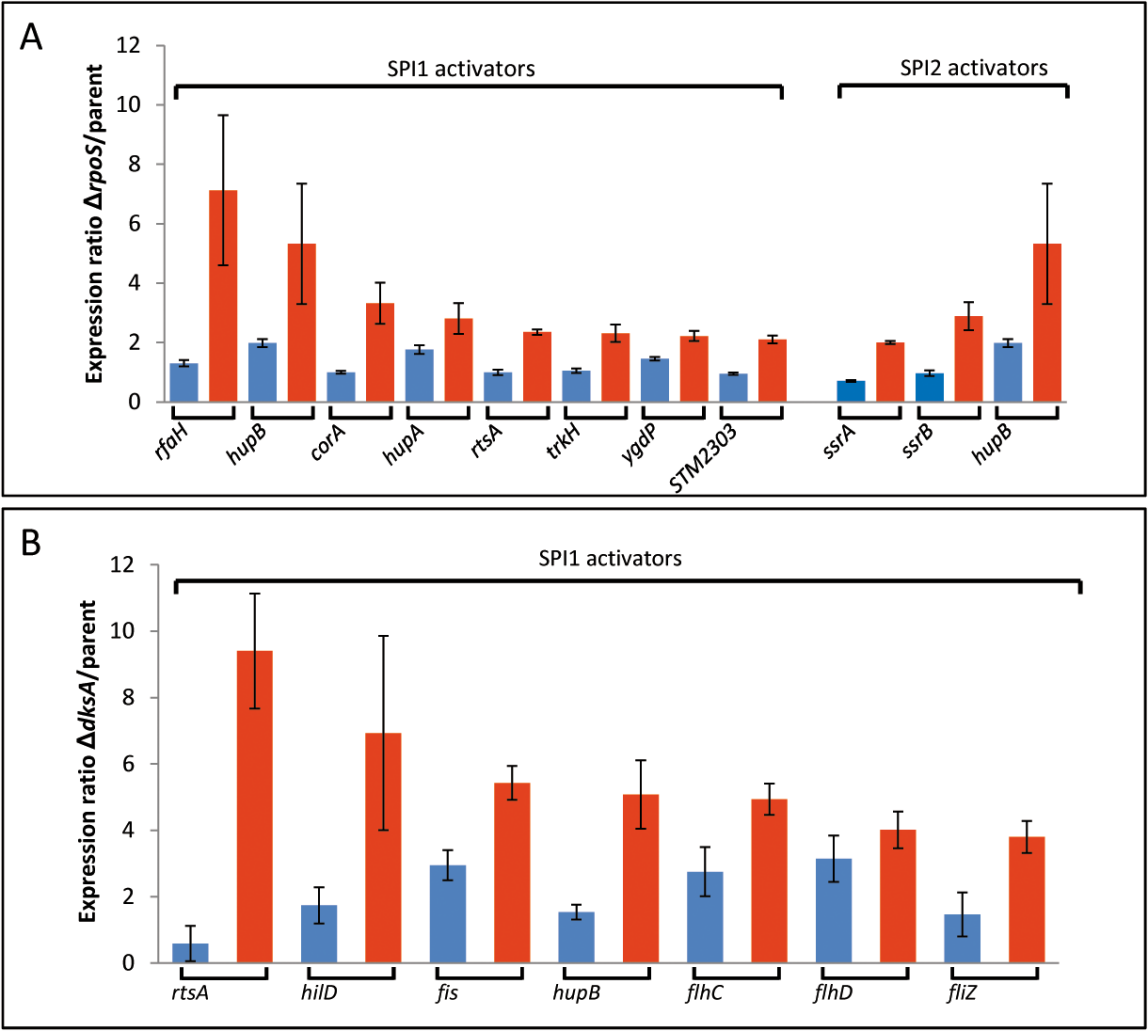
(A) Differential expression (> 2-fold) of known SPI1 and SPI2 activators at LSP and ESP in the Δ*rpoS* mutant normalised to the parent strain (*p* < 0.05). Blue and red bars correspond to ESP and LSP respectively [47,48]. (B) Differential expression (> 2-fold) of known SPI1 activators at LSP and ESP in the Δ*dksA* mutant normalised to the parent strain (*p* < 0.05). Blue and red bars correspond to ESP and LSP respectively. Data from which the figure was compiled and statistical analysis is shown in S3 Fig and also deposited at Gene Expression Omnibus (GEO), superseries accession number GSE63715.

The regulatory networks involved in the control of SPI1 and SPI2 genes are complex and operate at several levels [48]. So far, at least 65 and 23 regulators have been shown to be involved in the control of genes within SPI1 and SPI2, respectively [48,49]. To determine whether any of these regulators were transcriptionally RpoS and/or DksA-dependent, and therefore potentially involved in the regulation of SPI1 and SPI2 by RpoS and DksA, (in addition or instead of the proposed effects of sigma factor competition), we used microarrays to determine their expression levels in the Δ*rpoS* and Δ*dksA* mutants relative to the parent strains at ESP and LSP (Fig 6, S3 Table). A comparison of the known regulators of SPI1 at LSP compared to ESP showed significantly increased expression (> 2-fold) of several SPI1 activators in the Δ*rpoS* strain (Fig 6, S3 Table). The *rfaH* gene showed the greatest increase in expression between LSP and ESP in the Δ*rpoS* compared to the parent strain (7.1-fold and 1.3-fold at LSP and ESP respectively, Fig 6, S3 Table). An *rfaH* null mutation has been correlated with a 4-fold decrease in *hilA* expression under SPI1 inducing conditions in LB [48]. RfaH has also previously been shown to be modulated by RpoS in *S*. Typhi; in *S*. Typhimurium, the *rfaH* promoter also contains a predicted RpoS consensus sequence [7,50]. RfaH encodes a DNA-binding antiterminator, and is involved in the expression of distal genes in long, horizontally-acquired operons [51]. Its role in the regulation of SPI2 genes under conditions where SPI2 is expressed has not yet been determined; however, impaired intracellular replication within macrophages and mice has been demonstrated in an RfaH-deficient strain. This phenotype has been previously attributed to truncated LPS in *S*. Typhimurium [52]. Other RpoS-dependent activators which have been shown to increase both SPI1 and SPI2 expression and were significantly elevated > 2-fold at LSP compared to ESP in the Δ*rpoS* relative to the parent strain included *hupA*, *hupB*, *corA*, *rtsA*, *trkH*, *ydgP and STM2303* (S3 Table, Fig 6). Although *hilA* expression was increased by 2.4-fold at LSP in the Δ*rpoS* relative to the parent strain, it was not significant at *p* < 0.05 (S3 Table). Of the SPI2 activators, *ssrA*, *ssrB* and *hupB* were the most highly differentially expressed genes in the Δ*rpoS* compared to the parent strain at LSP compared to ESP. Expression of the *ssrA*, *ssrB* and *hupB* genes were significantly increased by 2.0, 2.9 and 5.3-fold respectively in the Δ*rpoS* compared to the parent strain at LSP, whereas at ESP their expression levels were 0.7, 1.0 and 2.0 respectively (Fig 6, S3 Table). The transcript levels of the *slyA* and *hupA* gene were also significantly overexpressed by 2.4, 2.8 fold respectively in the Δ*rpoS* compared to the parent strain at LSP (S3 Table), however, their transcript levels at ESP were 1.5 and 1.8-fold respectively. Therefore *ssrA*, *ssrB* and *hupB* displayed the highest ratio of transcript levels at LSP compared to ESP (Fig 6, S3 Table).

In the Δ*dksA* strain, SPI1 expression was elevated at LSP compared to ESP. A comparison of transcript levels for the known SPI1 regulators in the Δ*dksA* compared to the parent strain at LSP *vs*. ESP revealed significant differences in several known SPI1 activators. The activator showing the largest increase in expression at LSP compared to ESP in the Δ*dksA* relative to the parent strain was *rtsA*, (9.4-fold at LSP, S3 Table, Fig 6). RtsA is a major regulator of both *hilA* and *hilD* expression and forms part of a feed-forward loop for activation of SPI1 expression [53]. In addition, the expression of *hilD* was also significantly increased in the Δ*dksA* compared to the parent strain at LSP compared to ESP (6.9 and 1.7-fold respectively). Other significantly upregulated activators at LSP compared to ESP in the Δ*dksA* relative to the parent strain included *fis*, *hupB* and the genes encoding the flagellar regulators, *flhC*, *flhD* and *fliZ* (S3 Table, Fig 6). SPI2 gene transcript levels were reduced in the Δ*dksA* compared to the parent strain at all points sampled during late-log/stationary phase (Fig 1G). Of the known SPI2 activators, the expression of the major SPI2 activators *ssrA and ssrB* were significantly repressed between 2.2 and 6.1-fold in the Δ*dksA* relative to the parent strain at the four growth phases sampled (S3 Table). In addition, expression of *phoP* was reduced by 2.7-fold at both LLP and ESP and *phoQ* by 2.3 and 2.2 at LLP and MSP respectively in the Δ*dksA* relative to the parent strain (S3 Table). Expression of the SPI2 repressor, *ydgT* was also significantly increased by 2.3 and 2.7-fold at ESP and LSP in the Δ*dksA* compared to the parent strain (S3 Table). Which of these regulators are responsible for the RpoS and DksA-dependent alterations in SPI1 and SPI2 expression is the subject of future work.

## Discussion

In this study we show that RpoS, DksA and ppGpp play both antagonistic and complementary roles resulting in the modulation of SPI1 and SPI2 transcription during late-log and stationary phase growth in aerobic LB broth cultures. Whereas the absence of ppGpp resulted in decreased SPI1 encoded gene transcript levels at LLP, ESP, MSP and to a lesser extent at LSP (Fig 1B), in the Δ*rpoS* and Δ*dksA* strains, the transcript levels of most SPI1 encoded genes were increased at MSP and LSP (Fig 1B and 1C). The SPI1 genes showing the greatest increase in transcript levels in the Δ*rpoS* compared to the parent strain at MSP and LSP were members of the *sicA* operon. The SPI1 expression data for the Δ*rpoS*, Δ*dksA* and Δ*relA*Δ*spoT* strains therefore indicates that in the parent strain, ppGpp would be expected to activate the expression of most of the SPI1 encoded genes throughout stationary phase, (and to a lesser extent at LSP), whereas RpoS and DksA would have an antagonistic effect to ppGpp at MSP, and particularly at LSP, suggesting that the net result would be a modulation of SPI1 transcript levels such that their transcript levels were repressed during LSP compared to ESP and MSP. The loss of ppGpp did not reduce SPI2 transcript levels as highly compared to SPI1 transcript levels during LLP, ESP and MSP; however, at LSP, SPI2 transcript levels in the Δ*relA*Δ*spoT* strain were considerably reduced by up to 15-fold relative to the parent strain (Fig 1F). Similarly to SPI1, the absence of RpoS resulted in an increase in the majority of SPI2 transcript levels at MSP and LSP (Fig 1F); despite this, the differential effects of loss of ppGpp and RpoS on SPI2 transcript levels at LSP suggests that, in the parent strain, ppGpp is likely to play a significant role in elevating SPI2 transcript levels at LSP (Fig 1E). In a broader context, the effect of RpoS in reducing SPI1 and SPI2 transcript levels may partially contribute to the reason for the prevalence of *rpoS* mutations found in several *S*. Typhi field isolates, where they might be expected to enhance virulence traits [54]. In contrast to the marked growth phase specific effects of loss of ppGpp and RpoS on SPI2 gene transcript levels, the effect of loss of DksA was less obvious, and SPI2 transcript levels were reduced at LLP, ESP, MSP and LSP in the Δ*dksA* compared to the parent strain, suggesting DksA is required for SPI2 transcription throughout late-log/stationary phase (Fig 1G). The changing growth phase-dependent levels and/or activities of RpoS, ppGpp and DksA may also potentially be expected to have a significant impact upon the ‘timing’ of SPI1 and SPI2 expression during infection, since it is established that SPI1 and SPI2 genes tend to be expressed under conditions conducive to either invasion and intracellular replication respectively, although some overlap has been found [5].

It is of interest that, although DksA is synthesised constitutively throughout growth (Fig 4A, [20]), deletion of *dksA* resulted in opposing effects on SPI1 and SPI2 transcript levels during stationary phase (Fig 1C and 1G). The simultaneous activating and repressive effects of DksA on gene expression is not unprecedented [20,23,47,55]. DksA binds to RNAP and greatly enhances direct effects of ppGpp on the negative control of *E*. *coli* rRNA promoters [20]; in addition, DksA has also been shown to potentiate the direct activation of amino acid promoters by ppGpp [23]. DksA also has disparate effects on the expression of virulence determinants in *E*.*coli* 0157; although both ppGpp and DksA were required for activation of the *LEE1* promoter during entry into stationary phase, their effect was different at late stationary phase and *LEE1* promoter activity was increased in the Δ*dksA* strain [22]. These results and our own data indicate that DksA can have both positive and negative effects on the expression of different virulence genes in both *E*. *coli* O157 and *S*. Typhimurium; the exact mechanism by which this occurs remains to be clarified. However, recently a 5-bp AT rich discriminator region (P_dsc_, AAGGA), located immediately downstream of the -10 element has been shown to be critical for positive control of the *E*. *coli uspA* promoter by ppGpp/DksA [56]. SPI2 encoded genes are under the positive control of the major regulators SsrA/B, OmpR/EnvZ and SirA/BarA [57]. Neither of the two published transcriptional start sites (TSS’s) for the SPI2 *ssrA* promoters have a proximal upstream AAGGA motif, however the discriminator region immediately upstream of the *sirA* TSS contains an AAGGA motif: TAAGGA**G**, where **G** is the annotated TSS at genomic position 1996515 [43]. The *sirA* gene was found to be 2-fold repressed in the Δ*dksA* strain LSP compared to ESP (S3 Table), and therefore suggests a possible mechanism by which SPI2 may be indirectly activated by DksA. The *ssrA* promoter does however contain AT rich discriminator regions: ATTCTA**T** at genomic position 1436617 and TGTTGT**T** at genomic position 1436769 (where **T** represents the TSS; [43,58]). It remains to be seen whether these discriminator regions are directly involved in positive control by DksA/ppGpp.

The opposing effects of ppGpp and RpoS in respectively activating and reducing transcript levels of SPI1 and SPI2 genes during stationary phase and the observation that SPI1 and SPI2 gene transcript levels were not elevated in the Δ*relA*Δ*spoT*Δ*rpoS* strain compared to the Δ*rpoS* strain, yet remained at the same reduced levels observed in the Δ*relA*Δ*spoT* strain (Fig 1D and 1H) is in agreement with the ‘sigma factor competition’ model whereby ppGpp is required to facilitate competition between the sigma 70 ‘housekeeping’ factor and alternative sigma factors, based on their relative intracellular ratios [59]. In this model, ppGpp would be required for the recruitment of RNAP to SPI1 and SPI2 sites, whereas it would also facilitate competition between sigma 70 and RpoS, which would result in loss of RNAP from SPI1 and SPI2 promoter sites, perhaps contributing to the decreased SPI1 expression observed in the parent strain at LSP (Fig 1A). For SPI2, although RpoS is competing for recruitment of RNAP, ppGpp or ppGpp-dependent activating factors appear to play a role to facilitate the elevation of SPI2 transcript at LSP compared to earlier time points (Fig 1E). The sigma factor competition model would predict that overexpression of RpoS may result in reduced expression of the SPI1 effector gene *sipC*, due to the inferred redistribution of RNAP to RpoS-dependent promoter sites rather than SPI1 promoter sites and consistent with this, we confirmed that ectopic induction of RpoS resulted in severely reduced *sipC*::*lacZ* activity compared to the parent strain during stationary phase (Fig 5). Although the absence of SipC can be correlated with lack of *sipC* transcripts in the Δ*relA*Δ*spoT* strain, *sipC* transcript levels were increased in the Δ*rpoS* compared to the parent strain at MSP and LSP by 5.7 and 7.0 fold respectively (Fig 1B, S2 Table). In addition, activity of a *sipC*::*lacZ* fusion was increased 3-fold in the Δ*rpoS* background compared to the parent strain (Fig 3). Despite these observations, the elevated transcript levels appear to be modulated post-transcriptionally, resulting in little overall change in either the intracellular or secreted SipC protein levels (Fig 4A and 4B). This may suggest a mechanism whereby *sipC* and possibly other SPI1 encoded gene expression could remain ‘buffered’ against rapid changes in RNAP distribution caused by alternative sigma factors such as RpoS, thus potentially optimising a balance between the RpoS-dependent stationary phase stress response and invasion. The latter mechanism may also represent an adaptation by which limited RNAP availability can be efficiently managed to balance stress and virulence [59].

Similarly to RpoS, the absence of DksA also resulted in increased transcript levels of many SPI1 genes including *sipC* at MSP and LSP compared to the parent strain (Fig 1C). Deletion of *dksA* resulted in a slight reduction in RpoS (Fig 4A); however, since deletion of *rpoS* had no effect on SipC levels (Fig 4A), it seems unlikely that DksA is acting via RpoS to reduce parental *sipC* transcript levels. Despite the elevated *sipC* (and other SPI1) transcript levels in the Δ*dksA* strain at MSP and LSP, intracellular SipC protein remained completely undetectable throughout stationary phase in the Δ*dksA* mutant (Fig 4A). The latter result may explain the discrepancy between the observed elevation of SPI1 transcript levels in the Δ*dksA* strain and the invasion defect of the Δ*dksA* strain in HeLa cells invasion assays (S4 Fig). SipC was however present in the culture supernatants of both the Δ*dksA* and Δ*relA*Δ*spoT* strains, although at considerably reduced levels compared to the parent strain (Fig 4B); this may reflect SipC accumulated prior to late-log/stationary phase. The small reduction in overall activity of the *sipC*::*lacZ* fusion in the Δ*dksA* compared to the parent strain throughout stationary phase (Fig 3) and complete absence of SipC protein in the Δ*dksA* relative to the parent strain (Fig 4A) suggests that DksA is directly or indirectly required to stabilise SipC. Further experiments are in progress to determine the basis for the post-transcriptional regulation of the SipC effector protein by DksA.

Finally, a transcriptional analysis was performed to determine whether any of the known SPI1 and SPI2 activators could play a role in the observed differences in the transcript levels of SPI1 and SPI2 encoded genes at LSP in the Δ*rpoS* and Δ*dksA* mutants compared to the parent strain, as well or instead of any effects caused by potential sigma factor competition. We found that the most highly differentially expressed SPI1 activators at LSP compared to ESP were *rfaH* and *rtsA*. The transcript levels of *rfaH* in the ∆*rpoS* strain were increased by 5.5-fold at LSP compared to ESP, and *rtsA* transcript levels were increased by 15.9 fold in the Δ*dksA* strain at LSP compared to ESP (S3 Table). This may suggest that RpoS and DksA act via different regulatory pathways to efficiently repress SPI1 expression under the environmental conditions studied here. For SPI2, the transcript levels of the major SPI2 regulators *ssrA* and *ssrB* were the most highly differentially expressed genes in the Δ*rpoS* versus the parent strain at LSP compared to ESP, suggesting they may play a role in the observed elevation of SPI2 transcript levels at LSP (Fig 6).

## Materials and Methods

### Strains and culture conditions

A full list of strain details used in this study is described in S1 Table. *Salmonella enterica* sv. Typhimurium SL1344 parent and isogenic Δ*relA*Δ*spoT*, Δ*rpoS*, Δ*dksA*, Δ*relA*Δ*spoT*Δ*rpoS* and Δ*relA*Δ*spoT*Δ*dksA* strains were routinely grown in Luria-Bertani (LB) medium at 37°C, shaking at 250 rpm. For growth experiments, single colonies grown on LB agar were added to 5 mL LB and grown overnight, before inoculation into liquid LB medium (1:100). Strains were grown to optical densities (OD’s) measured at 600 nm corresponding to late-log phase (LLP, OD≈1.0), early stationary phase (ESP, OD≈2.3), mid-stationary phase (MSP, OD≈3.2) and late stationary phase (LSP, OD≈3.6), and samples taken at these points for further analysis. Optical density growth curves (OD_600_) and CFUs of sampling points are shown in S1 and S2 Figs. For experiments, requiring selection of strains or plasmids, antibiotics were added at the following concentrations: ampicillin (100 μg ml^-1^), chloramphenicol (20 μg ml^-1^), kanamycin (50 μg ml^-1^), and tetracycline (20 μg ml^-1^).

For experiments involving the controlled overexpression of *rpoS*, a DNA fragment containing the *rpoS* open reading frame (ORF) and *Eco*RI and *Bam*HI sites was cloned into the high copy number vector, pASK75 [58]. The *rpoS* ORF was amplified by PCR. The forward and reverse primer sequences (5’ to 3’), containing the restriction sites were TAGAGCGAATTCTAGGAGCCACCTTATGAGTC and CACCTTGGATCCCAAGGGTACTTACTCGCGGA respectively. After digestion with *Eco*RI and *Bam*HI, the fragment was ligated into the high copy number, inducible *tetA* ^p/o^, Amp^R^ vector, pASK75, which was transformed into four strains of *S*. Typhimurium (parent, Δ*rpoS*, Δ*dksA* and Δ*relA*Δ*spoT*) by electroporation. The plasmid was maintained by addition of 100 μg.ml^-1^ ampicillin to the culture medium and expression from the *tetA* promoter was induced by the addition of 0.2 μg.ml^-1^ anhydrotetracycline (ATC) (Fluka, 37919) as per the method outlined [60], and confirmed by western blotting.

### β-galactosidase assay

A *sipC*::Tn5*lacZY* transcriptional fusion from *S*. Typhimurium strain SA29 [61] was transduced by electroporation into four *S*. Typhimurium SL1344 genetic backgrounds (parent strain, Δ*rpoS*, Δ*dksA*, Δ*relA*Δ*spoT*) and used to assay *sipC* promoter activity. Strains containing the *sipC*::Tn5*lacZ* fusion were grown in batch cultures and the culture was sampled at 2h, 3h, 5h, 6h and 8h. For clarity, the growth curves and *sipC*::Tn5*lacZ* expression levels are shown in S3 Fig.

The β-galactosidase assay was performed at 28°C according to [62]. Briefly, 0.2 ml of cell culture was diluted in 0.8 ml Z-Buffer and 40 μl chloroform and 20 μl 0.1% sodium dodecyl sulphate added to permeabilise the cells. The reaction was initiated by the addition of *o*-nitrophenyl-β-D-galactoside (ONPG; 4 mg/ml). Once the reaction began to turn yellow, it was quenched by the addition of 0.5 ml 1M sodium carbonate. β-galactosidase activity was then measured spectrophotometrically at 550 nm and 420 nm, and the cell culture optical density measured at 600 nm. The data were expressed in Miller Units, according to the following equation: Miller Units = 1000 x [(OD_420_–1.75 x OD_550_)] / (T x V x OD_600_), where T = time of the reaction (minutes) and V = volume of culture used in the assay (ml).

### Microarray analysis

Microarray analysis was performed as described previously [43]. *S*. *Typhimurium* SL1344 parent and mutant strains were grown to LLP, ESP, MSP and LSP as described under Strains and culture conditions. Total RNA was extracted from the strains as described above. The RNA was labelled and hybridised to IFR SALSA2 microarrays (www.ifr.ac.uk/Safety/Microarrays/default.html#protocols), and data processed and analysed using GeneSpring (Agilent). The data was from 3 biological replicates, statistically filtered (*p* = 0.05) and a 2-fold cut off applied. The microarray data discussed in this publication are MIAME compliant and have been deposited in NCBI's Gene Expression Omnibus and are accessible through GEO accession number GSE63715.

### ChIP-chip analysis

Strains SL1344 parent and isogenic Δ*relA*Δ*spoT* and Δ*dksA* strains were grown in LB broth under normal aeration at 37°C to LLP, ESP, MSP, and LSP, as described under Strains and culture conditions. Co-immunoprecipitation was carried out using monoclonal antibody raised against the beta subunit of *E*. *coli* RNA polymerase (Neoclone, W0002) which has 100% sequence identity to *S*. Typhimurium RNAP. The CoIP protocol is described in [63]. Microarrays used for the ChIP-on-chip experiments were designed and produced by Oxford Gene Technology (Kidlington, UK). They consisted of approximately 44,000 60-mer oligonucleotides tiled throughout the *S*. Typhimurium SL1344 NCTC13347 genome and 636 control oligonucleotides giving a 100 nt resolution. Microarray hybridisations were carried out according to the manufacturer’s instructions. Further descriptions of the microarray and protocols used for generating and analysing the data are associated with the dataset deposited in the GEO data repository (www.ncbi.nlm.nih.gov/geo/) under accession number GSE63715. In order to identify peaks, the microarray data was loaded onto ChIPOTle Visual Basic for applications macro [64] and then visualized in the Integrated Genome Browser (IGB) Affymetrix (version 8.1.8), [65]. Each ChIP-chip experiment for each strain was performed in triplicate.

### Western blotting

To visualise intracellular bacterial protein production, parent and mutant strains were grown to LLP, MSP, ESP and LSP as described under Strains and culture conditions, harvested by centrifugation (7000×*g*, 4°C, 10 minutes) and re-suspended in 1× NuPAGE protein loading buffer (Life Technologies, NP0007), containing 50mM freshly-added dithiothreitol (DTT). For each 0.1 OD unit, 10 μl sample buffer was added. Lysis and solubilisation was carried out by boiling the samples for 10 minutes and centrifuging the lysates for 30 minutes (> 10,000×*g*). Lysates were diluted 1:10 and subjected to size separation by SDS-PAGE on 12% Bis-Tris Protein Gels (Life Technologies, NP0342PK2). Growth curves and optical densities at which samples were removed for western blotting are shown for parent, Δ*rpoS*, Δ*dksA* and Δ*relA*Δ*spoT* strains in S1 and S2 Figs.

Analysis of proteins in the cultures supernatant was performed using the method of [66]. Briefly, cell cultures were centrifuged to remove intact cells, and supernatants were passed through a 0.22μm filter. To 1 mL of supernatants, 0.3 ml ice-cold trichloroacetic acid was added and the samples left on ice for 15 min to precipitate the proteins. Samples were centrifuged at 10,000×*g* for 15 minutes, washed twice with acetone and the final protein pellet was dissolved in loading buffer and separated by SDS-PAGE.

After gel electrophoresis, proteins were transferred to a methanol-treated PVDF membrane using semi-dry transfer apparatus (Bio-Rad; 1h, 0.25A) and the membrane blocked using a solution of PBST containing 10% skimmed milk powder (Marvel). Antibodies to specific proteins (mouse α-RpoS (Neoclone, W0009; 1:1000), chicken α-DksA (1:2500), mouse α-SipC (1μg ml^-1^), rabbit α-GroEL (Sigma, G6532; 1:40000)) were added to binding solution (PBST + 0.5% Marvel) and incubated at room temperature, with vigorous agitation for 2 hours (primary antibody) or 1 hour (secondary antibodies). Detection of protein was performed using Goat IgG secondary antibodies, conjugated to horseradish peroxidase. Peroxidase activity was identified using Pierce SuperSignal West Pico Chemiluminescent Substrate (Thermo Scientific, 34080) and bands were visualised using the FluorChem E System (ProteinSimple). In order to ensure consistent loading, all blots were subsequently stripped according to the manufacturer’s instructions and re-probed with antibody against GroEL.

### Invasion assays

Invasion assays in HeLa epithelial cells (obtained from American Type Culture Collection, Rockville, MD) were performed according to [67]. Briefly, HeLa cells were grown in DMEM medium (Sigma, D5546) containing 1 g/L glucose and supplemented with 10% fetal bovine serum (Sigma), 2 mM L glutamine (Sigma) and 20 mM HEPES buffer (Sigma). Between 1 and 3 ×10^5^ HeLa cells were seeded into each well of a 6- or 12-well cell culture plate and infected with *S*. Typhimurium SL1344 and mutant strains at an MOI of 10:1. Prior to infection, the *S*. Typhimurium strains had been grown to an OD_600_ of 2.3 to allow expression of the SPI1 Type-3 secretion system. To increase the uptake of *Salmonella*, plates were centrifuged at 1000 g for 5 min, and this was defined as time 0 h. After 1 h of infection, extracellular bacteria were killed with 30 μg.ml^-1^ gentamicin. The media was replaced after 1 h with medium containing 5 μg.ml^-1^ gentamicin. Incubations were continued for 2 h and 6 h. To estimate the amount of intracellular bacteria at each time point, cells were lysed using 0.1% SDS, and samples were taken for viable counts. Statistical significances were assessed by using Student’s unpaired *t*-test, and a *p* value ≤ 0.05 was considered significant.

## Supporting Information

S1 Fig Growth phenotypes of SL1344 parental strain and Δ*rpoS*, Δ*relA*Δ*spoT* and Δ*dksA* strains in LB media and grown aerobically, with shaking (250 rpm) at 37°C. (DOCX)

S2 Fig Optical density and equivalent CFU’s at which samples were taken for protein extraction and western blot analysis, shown in Fig 4. (DOCX)

S3 Fig Growth characteristics and LacZ activities for samples assayed for *sipC* promoter activity shown in Fig 3. (DOCX)

S4 Fig Invasion assay of *S*. Typhimurium SL1344 parental and Δ*dksA* strain in HeLa cells. (DOCX)

S1 Table Strains and plasmids used in this study. (DOCX)

S2 Table Expression levels and statistical analysis of SPI1 and SPI2 encoded genes in parent and mutant strains used in the construction of Fig 1. (XLSX)

S3 Table Expression levels and statistical analysis of known SPI1 and SPI2 activators and repressors in parent and mutant strains used in the construction of Fig 6. (XLSX)
