# Supplementary material for: Transcriptional and Post-Transcriptional Modulation of SPI1 and SPI2 Expression by ppGpp, RpoS and DksA in Salmonella enterica sv Typhimurium
Source: PLoS One. 2015 Jun 3;10(6):e0127523. doi: 10.1371/journal.pone.0127523 (PMC4454661; doi:10.1371/journal.pone.0127523)
Supplement: S1 Fig — (DOCX) [file pone.0127523.s001.docx]

Parent

Δ*dksA*

Δ*rpoS*

Δ*relA*Δ*spoT*

**Figure S1.** Growth curves of parent and mutant strains in aerobic LB batch cultures shaken at 250 rpm in an orbital water bath set at 37^°^C. Cultures were inoculated with single colonies picked from LB agar plates streaked with strains from frozen stock and grown overnight at 37^°^C.
