# Supplementary material for: Transcriptional and Post-Transcriptional Modulation of SPI1 and SPI2 Expression by ppGpp, RpoS and DksA in Salmonella enterica sv Typhimurium
Source: PLoS One. 2015 Jun 3;10(6):e0127523. doi: 10.1371/journal.pone.0127523 (PMC4454661; doi:10.1371/journal.pone.0127523)
Supplement: S2 Fig — (DOCX) [file pone.0127523.s002.docx]

**Figure S2.** Optical densities and equivalent CFUs at which samples were removed for protein extraction for Western blot analysis shown in Fig. 4 in the main text. LL = late log phase, ES = early stationary phase, MS = mid-stationary phase, LS = late stationary phase. (A) SL1344 parent strain. (B) SL1344 Δ*rpoS*. (C) SL1344 Δ*dksA*. (D) SL1344 Δ*relA*Δ*spoT*. Experimental details are described in ‘Methods’ in the main text.
