# Supplementary material for: Transcriptional and Post-Transcriptional Modulation of SPI1 and SPI2 Expression by ppGpp, RpoS and DksA in Salmonella enterica sv Typhimurium
Source: PLoS One. 2015 Jun 3;10(6):e0127523. doi: 10.1371/journal.pone.0127523 (PMC4454661; doi:10.1371/journal.pone.0127523)
Supplement: S3 Fig — (DOCX) [file pone.0127523.s003.docx]

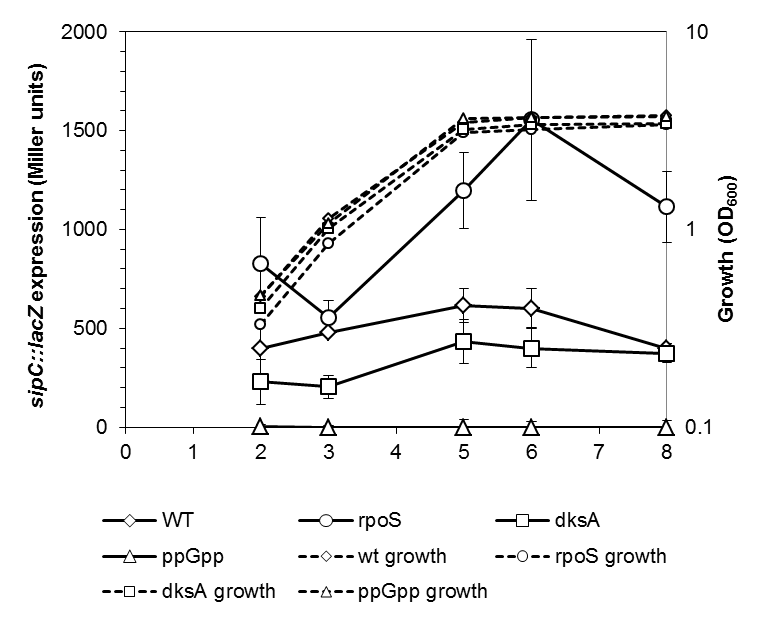


**Figure S3**. Growth characteristics and LacZ activities for samples assayed for *sipC* promoter activity shown in Fig. 3 in the main text.
