# Supplementary material for: Transcriptional and Post-Transcriptional Modulation of SPI1 and SPI2 Expression by ppGpp, RpoS and DksA in Salmonella enterica sv Typhimurium
Source: PLoS One. 2015 Jun 3;10(6):e0127523. doi: 10.1371/journal.pone.0127523 (PMC4454661; doi:10.1371/journal.pone.0127523)
Supplement: S4 Fig — (DOCX) [file pone.0127523.s004.docx]

***

***

**Figure S4.** Invasion assay of *S*. Typhimurium SL1344 parental and Δ*dksA* strain in HeLa cells. Light and dark grey shading corresponds to parent and Δ*dksA* strains respectively. Each bar represents the statistical mean from three biological replicates and the error bars represent the standard deviation. (The significant differences between the parental and the Δ*dksA* strains are shown by asterisks, *p,0.05. **p,0.01, and ***p,0.001).
