# Supplementary material for: Transcriptional and Post-Transcriptional Modulation of SPI1 and SPI2 Expression by ppGpp, RpoS and DksA in Salmonella enterica sv Typhimurium
Source: PLoS One. 2015 Jun 3;10(6):e0127523. doi: 10.1371/journal.pone.0127523 (PMC4454661; doi:10.1371/journal.pone.0127523)
Supplement: S1 Table — (DOCX) [file pone.0127523.s005.docx]

**Table S1** Strains and plasmids used in this study.

| ***S.* Typhimurium strain designation** | **Relevant genotype** | **Method of construction** | **Reference** |
| --- | --- | --- | --- |
| SL1344 | *hisG46 rpsL* | N/A | [1] |
| KT4514 | SL1344 *hisG46 rpsL* ∆*relA*∆*spoT* | λ Red mutagenesis [2] | Kind gift from Dr. Karsten Tedin (Freie Universität, Berlin) |
| JH3364 | SL1344 *hisG46 rpsL* Δ*rpoS*::Cm | λ Red mutagenesis | This work |
| AT1046 | SL1344  *hisG46 rpsL* Δ*dksA*::Cm | λ Red mutagenesis | This work |
| JH3395 | SL1344 *hisG46 rpsL* ∆*relA*∆*spoT*Δ*rpoS*::Cm | λ Red mutagenesis | This work |
| AT1047 | SL1344 *hisG46 rpsL* ∆*relA*∆*spoT*Δ*dksA*::Cm | λ Red mutagenesis | This work |
| JH3123 | SL1344 *hisG46 rpsL*  *sipC*::Tn5*lacZY* | P22 transduction from SA29 [3] | This work |
| AT1347 | SL1344 *hisG46 rpsL* Δ*rpoS*::Cm  *sipC*::Tn5*lacZY* | P22 transduction | This work |
| AT1348 | SL1344 *hisG46 rpsL* Δ*dksA*::Kan  *sipC*::Tn5*lacZY* | P22 transduction | This work |
| AT1349 | SL1344 *hisG46 rpsL* ∆*relA*∆*spoT*  *sipC*::Tn5*lacZY* | P22 transduction | This work |
| AT1350 | SL1344 *hisG46 rpsL* *sipC*::Tn5*lacZY* (pASK75) | P22 transduction | This work |
| AT1351 | SL1344 *hisG46 rpsL sipC*::Tn5*lacZY* (pASK75 *rpoS*+) | P22 transduction | This work |
| **Plasmids** |  |  |  |
| pASK75 | Ap^R^ high-copy-number vector, with inducible TetRA promoter. | N/A | [4] |
| pASK75::*rpoS* | Ap^R^ high-copy-number vector, with inducible TetRA promoter, expresses *rpoS*. | N/A | [4] |

1. Wray C, Sojka WJ (1978) Experimental *Salmonella typhimurium* infection in calves. Res Vet Sci 25: 139-143.

2. Datsenko KA, Wanner BL (2000) One-step inactivation of chromosomal genes in *Escherichia coli* K-12 using PCR products. Proc Natl Acad Sci U S A 97: 6640-6645.

3. Akbar S, Schechter LM, Lostroh CP, Lee CA (2003) AraC/XylS family members, HilD and HilC, directly activate virulence gene expression independently of HilA in *Salmonella typhimurium*. Mol Microbiol 47: 715-728.

4. Skerra A (1994) Use of the tetracycline promoter for the tightly regulated production of a murine antibody fragment in *Escherichia coli.* Gene 151: 131-135.
